# High-speed swept source optical coherence Doppler tomography for deep brain microvascular imaging

**DOI:** 10.1038/srep38786

**Published:** 2016-12-09

**Authors:** Wei Chen, Jiang You, Xiaochun Gu, Congwu Du, Yingtian Pan

**Affiliations:** 1 Department of Biomedical Engineering, Stony Brook University, Stony Brook, NY 11794, USA; 2 Key Laboratory of Developmental Genes and Human Diseases, Department of Anatomy and Neuroscience, School of Medicine, Southeast University, Nanjing 210009, China

## Abstract

Noninvasive microvascular imaging using optical coherence Doppler tomography (ODT) has shown great promise in brain studies; however, high-speed microcirculatory imaging in deep brain remains an open quest. A high-speed 1.3 μm swept-source ODT (SS-ODT) system is reported which was based on a 200 kHz vertical-cavity-surface-emitting laser. Phase errors induced by sweep-trigger desynchronization were effectively reduced by spectral phase encoding and instantaneous correlation among the A-scans. Phantom studies have revealed a significant reduction in phase noise, thus an enhancement of minimally detectable flow down to 268.2 μm/s. Further *in vivo* validation was performed, in which 3D cerebral-blood-flow (CBF) networks in mouse brain over a large field-of-view (FOV: 8.5 × 5 × 3.2 mm^3^) was scanned through thinned skull. Results showed that fast flows up to 3 cm/s in pial vessels and minute flows down to 0.3 mm/s in arterioles or venules were readily detectable at depths down to 3.2 mm. Moreover, the dynamic changes of the CBF networks elicited by acute cocaine such as heterogeneous responses in various vessel compartments and at different cortical layers as well as transient ischemic events were tracked, suggesting the potential of SS-ODT for brain functional imaging that requires high flow sensitivity and dynamic range, fast frame rate and a large FOV to cover different brain regions.

Optical coherence tomography (OCT) has emerged as an enabling biophotonic imaging modality for preclinical research and for clinical diagnostics^1^,^2^. Advances in OCT techniques such as optical Doppler tomography (ODT) and optical coherence elastography extend the OCT imaging capability from biological tissue morphology to blood flow dynamics and biomechanical properties, which will allow for more comprehensive understanding of the biological system physiology and function^3^,^4^. Recent applications of ODT and optical coherence angiography (OCA) to rodent brain imaging have shown great promise for visualizing 3D cerebrovasculature and probing cerebral blood flow (CBF) networks and thus neurovascular hemodynamics in the brain without the need for fluorescence tracker^5^,^6^. While OCA provides superb sensitivity and fast acquisition for imaging cerebral vasculature, ODT enables quantitative imaging of 3D CBF velocity (i.e., CBF*v* or red blood cell velocity, *v*RBC) networks, namely, directional flow rates that are highly desirable and well correlate with the neurovascular events in the brain^7^. Spectral-domain OCT (SD-OCT) - referred herewith as camera-based SD-OCT has been the prime option for ODT implementation owing to its superb phase stability and the advent of various ultra-broadband light sources. Ultrahigh-resolution SD-ODT (μODT) has been reported to image capillary flows and their dynamics changes in the mouse cortex^8^. However, due to technical limitations (e.g., frame rate, spectral resolution), SD-ODT may be prevented from applications where high temporal resolution and deep image depth are desired^9^,^10^.

On the other hand, recent advances in swept-source-based SD-OCT (SS-OCT) have shown great promise for *in vivo* 3D volumetric imaging in near real time and with high signal-to-noise ratio (SNR) roll-off^11^,^12^. New sources with broader-band and faster spectral sweeping markedly enhances the spatial (i.e., axial) and temporal resolutions SS-OCT^13^ to potentially image microvascular flows with higher dynamic range^10^. However, phase noises from swept source instability (e.g., trigger and clock jitters) may jeopardize Doppler signaling, especially for probing slow microvascular flows or acquiring at high frame rate which dwindles flow-induced Doppler phase shift^14^. Several techniques were reported to compensate phase jittering noise in SS-ODT, such as by implementing common-path schemes with one mirror to minimize phase errors near a certain depth^15^,^16^ or double mirrors for multiple depths after phase unwrapping^14^. These methods required sophisticated hardware modification and intensive computation. A recent interesting method used a fiber Bragg grating (FBG) in one of the balanced detection arms to track each spectral sweeping and thus correct the phase jittering between A-scans for ODT reconstruction^17^. Although much simpler, this method involved accurately matching the pathlengths between the two arms, but improved OCA image quality was shown^18^,^19^.

In this paper, a simple method was reported to effectively improve the phase stability of SS-ODT. A FBG filter was introduced between the swept source and the input arm of the OCT engine, thus facilitating a spectral-hopping mode (i.e., zeroing spectral output within the narrow FBG band) to calibrate the sweep trigger signal of the light source. Then, cross-correlation was applied to accurately register and thus compensate phase triggering errors among the A-scans. Combined structural and flow mask in motion estimation was implemented to further reject the random phase fluctuations and major phase shifts within large fast flows. GPU accelerated programming was applied to facilitate big data/image processing and real-time 3D flow display during image acquisition. The system performance was first quantified in flow phantom study and then validated in *in vivo* experiment, in which 3D cerebrovasculature and quantitative CBF networks of mouse cortical brain over a large field of view (FOV: 8.5 × 5 × 3.2 mm^3^) were acquired through a thinned-skull cranial window. In addition, brain functional changes such as the CBF network dynamics in response to a pharmacological challenge (e.g., acute cocaine administration) was acquired^20^, which clearly demonstrated the capability of our SS-ODT to enable 3D quantitative imaging of 3D blood flow network dynamics with markedly improved flow detection sensitivity, at high spatiotemporal resolutions, and over a large FOV.

## Results

### Flow sensitivity characterization with flow phantom

**Figure 1:**
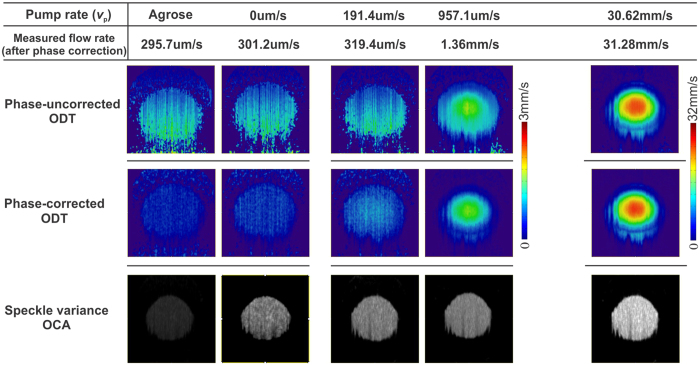
Comparative results of phase-uncorrected ODT, phase-corrected ODT and speckle-variance OCA images at different flow velocities (0.5% intralipid with 1.5% agarose for background quantification; 0.5% intralipid suspension at *v*_p_ = 0-, 191.4-, 957.1-μm, and 30.62 mm/s for flow sensitivity quantification).

For most *in vivo* scenario with low SNR, speckle noise resulting from multiple scattering and Brownian motion may contribute to phase noises and thus further elevate the minimally detectable flow. A phantom study was performed to mimic blood flows in brain vessels using 0.5% intralipid in a micro tube (PE10, ID = 280 μm). A high precision syringe pump was used for accurate flow rate control. Phase subtraction method (PSM)^21^ and speckle variance method^22^ were used to reconstruct ODT and OCA images, respectively. Figure 1 compares the ODT images before and after phase correction for pump flow rates *v*_p_ = 0-, 191.4-, 957.1-μm, and 30.62 mm/s (high pump flow to characterize flow dynamic range). Note that the background flow noise limit (first column) was quantified in solidified phantom by mixing 0.5% intralipid with 1.5% agarose. The ODT images before phase correction show predominant noise patterns (913.2 μm/s) induced by timing jitter, which increases with depth as predicted; after phase correction, the noise flow is dramatically reduced to 295.7 μm/s and appears uniform with depth. Similarly, the OCA image shows almost no noise flow (σ_OCA_ = 4k counts). In 0.5% intralipid suspension with no pump flow (*v*_p_ = 0 mm/s), the flow noise floor of ODT after phase correction is very low (301.27 μm/s) and comparable to the noise background (295.7 μm/s), but that of OCA is significantly increased to σ_OCA_ = 12 k counts due to non-directional Brownian motion. In contrast, due to overwhelming phase noise, the ODT images without phase correction fail to differentiate minute flow increase until *v*_p_ = 957.1 μm/s. The comparison clearly shows that our phase correction approach can effectively enhance the detection of minimal flow rate from 957.1 μm/s without phase correction down to 301.2 μm/s, which is crucial for quantitative imaging of blood flow in biological systems, such as cerebral blood flows for brain functional studies. On the other hand, in the high flow regime, e.g., *v*_p_ = 30.62 mm/s, both phase corrected and uncorrected ODT images start to saturate, thus demonstrating the advantage of 200 kHz SS-ODT for enabling a high dynamic range to detect fast flows.

### 3D SS-ODT for quantitative CBF imaging of mouse brain over a large field of view

**Figure 2:**
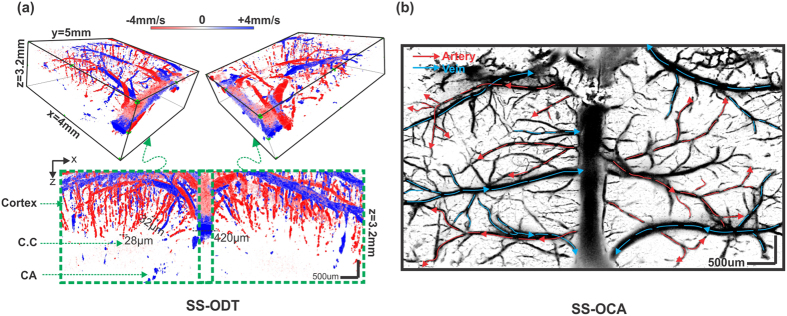
(**a**) Upper panel: 3D SS-ODT acquired at 200 k A-scan rate to show quantitative microcirculatory CBF networks in mouse brain over a large field of view (FOV: 8.5 × 5 × 3.2 mm^3^) thru thinned skull, lower panel: a 2D cross-sectional SS-ODT image across both hemispheres of mouse brain down to 3.2 mm of depth (i.e., including both cortex and hippocampus). Apparent flow directions are separated with respect to Doppler phase shifts and coded as red for descending and blue for ascending flows. (**b**) The corresponding SS-OCA image with red arrows to represent arterial compartments and blue arrows to represent venous compartments, segmented according to flow directions defined by SS-ODT. Deep microvascular flows ranging from 420 μm to 28 μm were readily detectable. CC: corpus callosum, CA: hippocampus.

We performed *in vivo* mouse brain study to demonstrate the utility of our newly developed SS-ODT system for quantitative imaging of the CBF networks. In this study, a large area over 8.5 × 5 mm^2^ across both left and right hemispheres of a mouse cortex was created by thinning the skull thru which 3D SS-ODT scans at 200 kHz A-scan rate were acquired within 80 s. Each B-scan contained 16 k A-scans, yielding 19.2-times oversampling across an 8.5 mm image window (x-axis) with 10.2 μm of transverse resolution. 1000 B-scans were sequentially acquired across 5 mm along y-axis to form a 3D stack containing 32.8 G voxels. Figure 2a shows the result of 3D SS-ODT of quantitative CBF networks in mouse over a large field of 8.5 × 5 × 3.2 mm^3^. Increased frame rate of SS-ODT facilitated enhanced dynamic range to detect fast flows up to ~3 cm/s for major vessels such as superior sagittal sinus and slow minute flows down to 0.3 mm/s in arterioles or venules. By combining distinct flow directions (e.g., descending flows in red and ascending flows in blue) and their vascular hierarchies^23^, arteries and veins are segmented as arterial vessels by red arrows and venous vessels by blue arrows. Although less microcirculatory flows (e.g., capillary flows) were detected than 3D μODT due to limited spatial resolution and sensitivity, the side-view image readily shows the penetrating arterioles and venules and the connecting terminal flows to a great depths up to 3.2 mm below the pial surface to the layers such as the corpus callosum (CC) and even the hippocampus (CA). These layers play critical roles in neurovascular hemodynamics and are thus of high relevance to studying brain function.

In parallel, the SS-OCA image was obtained by 8 repeated B-scans and reconstructed based on speckle variance approach^24^ to derive 3D cerebrovasculature, as shown in Fig. 2b. Although SS-OCA is unable to quantify flow rates, they provide high sensitivity and SNR to image vascular networks such as detailed venous and arterial branches whose flow directions are highlighted by blue and red arrows, respectively.

### Functional imaging of CBF dynamics using high-speed SS-ODT

**Figure 3:**
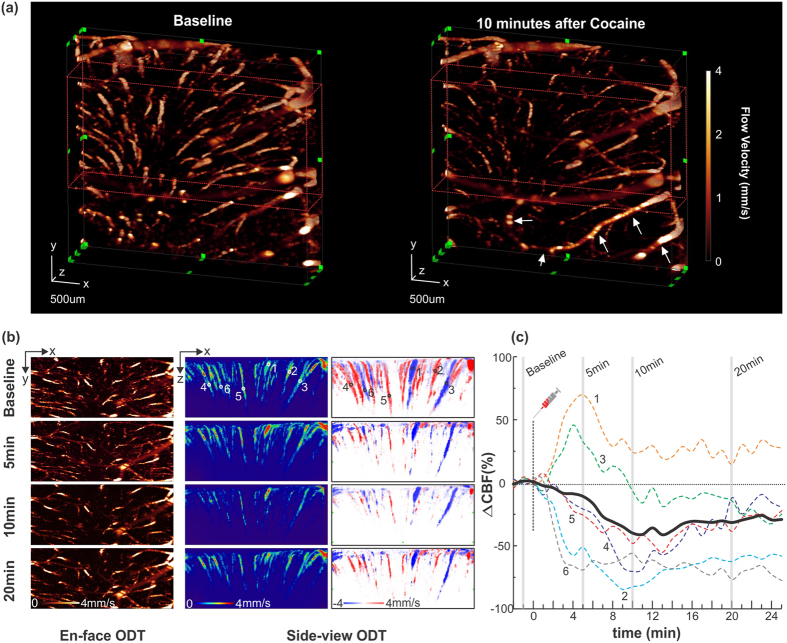
(**a**) Dynamic changes of 3D CBF networks (FOV: 3 × 3 × 1.8 mm^3^) in mouse somatosensory cortex in response to acute cocaine (30 mg/kg, *i.p.*), (**b**) En-face and cross-sectional projections to show the flow dynamics in different vessels (e.g., 1–6) after cocaine, (**c**) Time-lapse CBF change (ΔCBF%) to track the dynamics of different vascular compartments (e.g., arterioles and venules) in response to cocaine.

In addition to quantitative 3D imaging of CBF networks, high-speed SS-OCT permits functional study of brain hemodynamic changes such as the 3D CBF dynamics in response to brain activations with high temporal resolution. We examined the utility of our SS-ODT for functional imaging of the CBF network dynamics in response to an acute cocaine challenge. 3D SS-ODT was performed in the mouse sensorimotor cortex (3 × 3 mm^2^) before and after acute cocaine (30 mg/kg, *i.p.*). Figure 3a shows 3D SS-ODT images acquired at baseline and the flow decrease at 10 min after cocaine. Since each 3D image was acquired in less than 40 s (i.e., 16 k A-scans per B-scan, 500 B-scans total), rapid SS-ODT (200 kHz) enabled us to observe transient CBF changes elicited by cocaine over a large field of view. For instance, while CBF in most vessels dramatically decreased (e.g., >50%), it increased in some vessels as indicated by arrows in panel (Fig. 3a), which reflects the heterogeneous nature of the CBF networks to cocaine effects. Interestingly, the high temporal resolution of SS-ODT allowed to differentiate the timing sequences of the CBF changes in different vessels, as shown in Fig. 3b. For instance, flows in terminal vessels in deep cortex started to decrease within the first 5 min after cocaine injection, followed by a global flow decreases including in the larger vessels in the upper cortex and partial recovery after 20 min. Figure 3c plots the relative CBF changes (∆CBF%) in 6 vessels of different types (e.g., arterioles and venules) located at different cortical depths to track the CBF dynamics. While the averaged flow rate decreased 40.35% at 10 min after cocaine, some vessels (1 and 3) increased first (1~4 min after cocaine) and then decreased. Such imaging ability to differentiate CBF dynamics can be of interest for understanding complex relationship between brain function, behavior and CBF dynamics across different cortical layers and regions as well as in different vascular trees.

## Discussion

We reported the development of high-speed SS-ODT based on 200 kHz VCSEL swept source and GPU-accelerated GUI to implement real-time 3D flow image reconstruction and display. Phase errors induced by sweep trigger signaling (i.e., instability of A-scan synchronization) - a major problem hindering SS-OCT for Doppler flow detection – were effectively reduced by use of a FBG filter in the input arm of the OCT engine to calibrate the sweep trigger signal of the swept source. Unlike previous approaches that inserted a FBG in the one arm of the balanced detectors that generated a saturated spectral spike, this method operating in spectral-hopping mode provide a spectral zeroing output within the narrow FBG band, which allowed us to implement cross-correlation to accurately register and thus compensate phase triggering errors among the A-scans. Compared with previous methods (e.g., threshold method), the cross-correlation method shows increased phase correction accuracy and thus decreased phase noise level (Fig. s1), resulting in enhanced flow detection sensitivity as demonstrated in the flow phantom study (Fig. s2). Previous intensity thresholding method relied on a pre-defined threshold within the FBG band for phase error correction. This threshold criterion to pinpoint the timing of the turning wavelength could be disturbed by intensity noise from paired photodetector or the intensity fluctuation induced by the scattering sample. In comparison, the proposed method calculates the cross-correlation among multiple spectral points (e.g., n = 30–50 unsaturated signals) within the FBG band instead of a single turning wavelength, it is therefore less venerable to intensity noise and fluctuation, resulting in more robust and accurate phase correction. Moreover, the proposed method avoids the complications of previous methods such as dispersion mismatch and parasitic light reflection and thus reduced dynamic range due to overflow signals within the FBG band. All these advantages greatly enhance SS-ODT for effective phase correction and make it readily applicable to imaging flows in various biological tissues no matter whether there are strong or weak surface reflections or backscattering. System characterization studies showed that the overwhelming phase jittering noise (68mrad) was effectively reduced by 73.5% to 18mrad after phase correction. This corresponded to an enhancement of minimally detectable blow from 1013.2 μm/s to 268.2 μm/s, which approached to the estimated theoretical flow detection limit (254 μm/s, with OPD = 500 μm and SNR = 106.3 dB). Flow phantom study showed consistent results that the measured background flow noise (913.2 μm/s) was markedly reduced (67.6%) to 295.7 μm/s, which fell below the Brownian-motion-limited flow detection level of 301.27 μm/s. *In vivo* validation studies were performed to image mouse cerebrovascular networks over a large field of view (8.5 × 5 × 3.2 mm^3^) through a cranial window with thinned intact skull. With GPU-accelerated image real-time processing, high-speed SS-ODT imaging of 3D quantitative CBF networks was achieved with real-time 3D flow image reconstruction and display at a volume rate of 80 s per cubic image. With fast frame rate and improved flow detection sensitivity that facilitated enhanced dynamic range, the microcirculatory CBF networks, including both fast flows up to ~3 cm/s in major vessels and slow minute flows down to 0.3 mm/s in 28 μm arterioles or venules, were readily detectable at depths down to 3.2 mm in mouse brain (e.g., hippocampus). Such a fast flow imaging capability enables us to track brain functional changes such as CBF dynamics in response to acute cocaine challenge, e.g., transient ischemic events and heterogeneous responses in various vessel compartments and at different cortical layers. These results suggest that the new SS-ODT technique may find applications in brain functional studies such as brain connectivity studies that require high flow sensitivity and dynamic range, fast frame rate and to large field of view to across different brain regions. We notice that less capillary CBF network was imaged than μODT^8^, possibly due to limited spatial resolution of the current SS-ODT system (e.g., >10 μm), and we are working on optical and opto-mechanical designs to overcome the limitation.

## Methods

### 200 kHz swept-source OCT based on vertical-cavity surface-emitting laser

**Figure 4:**
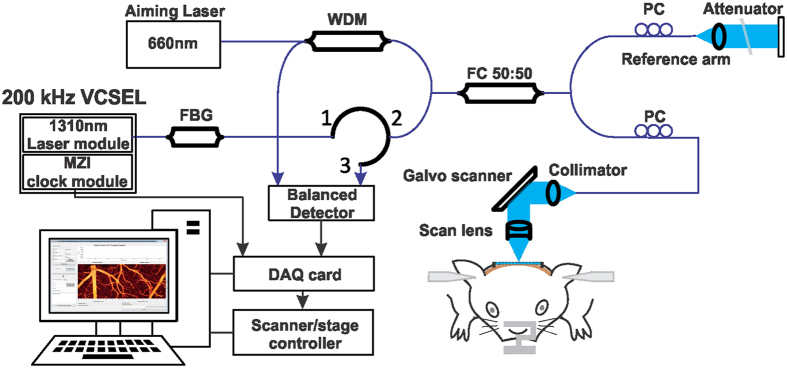
Schematic diagram of the SS-OCT system. VCSEL: vertical-cavity surface-emitting laser; MZI: Mach-Zehnder interferometer; FBG: fiber Bragg grating (λ_0_ = 1270.4 nm, reflectivity = 99.91%, ∆λ = 0.4 nm); WDM: wavelength-division multiplexing module; PC: fiber polarization controller; DAQ: data acquisition; FC: fiber coupler.

The high-speed SS-OCT system developed in our lab is illustrated in Fig. 4, which was powered by a MEMS-based VCSEL tunable laser engine (SL1310V1-20048, Thorlabs, NJ) capable of linearly sweeping across a broad wavelength range over 104 nm (at −10dB) from 1260 nm to 1364 nm at 200 kHz. The output power was 26.1 mw with 71% unidirectional swept duty cycle. The pigtailed laser was connected to a fiber Bragg grating (FBG) (central wavelength λ_0_ = 1270.4 nm, reflectivity R = 99.91%, laser linewidth ∆λ = 0.4 nm; OE Land, Quebec, Canada) before it was launched to illuminate a custom fiber optic interferometer consisting of a circulator (CIR), a 50/50 fiber coupler that split light into the reference and sample arms. Light exiting the sample arm was collimated Ø4 mm, transversely steered by a Galvo mirror (Cambridge instrument, MA), and then focused onto the sample using a 5 ×  NIR scan lens (EFL = 36 mm, Thorlabs, NJ), yielding a focal spot size (i.e., lateral resolution) of 14.3 μm in air or ~10.2 μm in biological tissue. In the reference arm, a titled neutral density filter was place in front of a gold-coated mirror mounted onto a translational stage to match the light density and pathlength in sampling arm. Reflected light beams from both the sample and the reference arms were recombined in the detection arms where the interference fringes were detected using a balanced photo detector with a bandwidth of DC~350 MHz (PDB130C, Thorlabs, NJ) and then digitized using a 12 bit, 1.8GS/s data acquisition card (ATS9360, AlazarTech, QC, Canada). The k-clock was generated by a delayed Mach-Zehnder interferometer (MZI) module and connected to the data acquisition card as sampling clock to allow accurate depth-profile decoding (iFFT) for Nyquist-sampling limited full depth imaging (e.g., >6 mm); image acquisition and transverse laser scan were synchronized to the spectral sweep trigger signal from the laser, which is crucial for phase-based ODT or OCA imaging. An aiming laser at 660 nm was launched into the OCT engine through the WDM to align the sample in the experiment.

### Graphic processing unit (GPU) accelerated real-time ODT reconstruction and display

**Figure 5:**
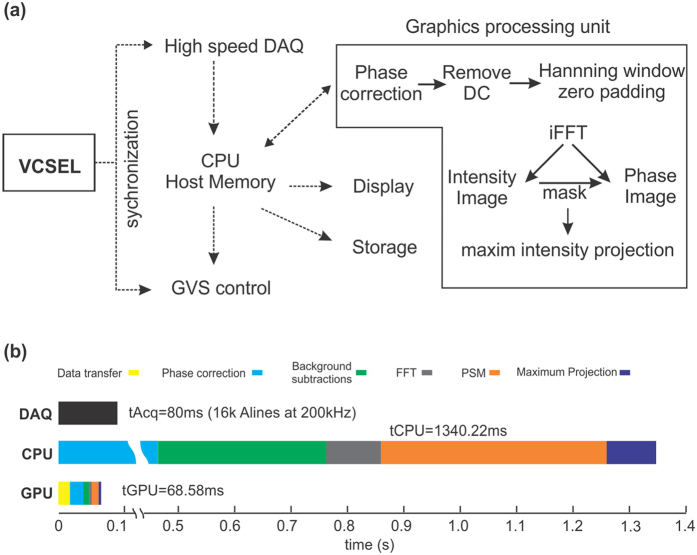
(**a**) Flow chat of SS-OCT data acquisition and parallel Doppler flow image reconstruction with GPU, (**b**) Improvement chart for accelerating parallel image reconstruction with GPU. DAQ: data acquisition, GVS: galvo scanner, iFFT: inverse fast Fourier transform.

To facilitate *in vivo* studies, we customized a GPU-accelerated graphic user interface (GUI), which was implemented in multi-threads using C++ to accommodate the demand for handling high data throughput (up to 8Gbits/s) and intensive calculation needed for real-time image reconstruction. Parallel computing algorithm was programmed in GPU (CUDA, NVIDIA) to facilitate real-time visualization of blood flow dynamics. Figure 5a illustrates the flow chat for data acquisition and processing. Ultrafast data acquisition was synchronized with transverse light scanning via MZI’s k-clock and sweep trigger signals of the VCSEL source. Each B-scan was transferred through computer CPU to GPU for image processing, where phase error was corrected first before zero-padding and then iFFT was executed to extract both intensity (OCT) and phase (ODT) images. Intensity mask was generated to reject random noise in the phase image. Both current cross-sectional 2D flow image and instantaneously updating 3D en-face image were displayed during image acquisition. Figure 5b compares the computation time usage, showing dramatic improvement with GPU acceleration. For example, for a typical B-scan image containing 16,000 A-lines with 2860 spectral sampling points, a serial algorithm implemented by CPU (i7-3820, Intel) took 1340.22 ms to complete the calculation; whereas by parallel computing with GPU acceleration (GeForce GTX670, NVIDIA), it was reduced to 68.58 ms to fulfill the same task, which significantly enhanced background calibration and phase subtraction processes. Since a B-scan at 200 kHz A-line rate only needs 80 ms, >1 s computation time with CPU exceeds the data acquisition time; with GPU acceleration, the computation time (68.58 ms) is reduced to be shorter than image acquisition (<80 ms), thus complying by the computing time budget of the SS-ODT system for real-time visualization of flow dynamics.

### Cross-correlational interferogram registration to enable accurate phase correction

**Figure 6:**
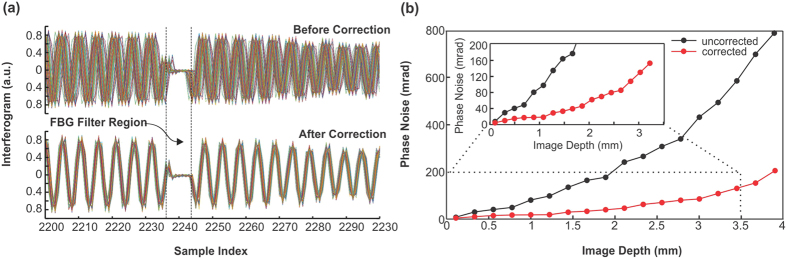
(**a**) Overlapped interferograms from successive A-scans before and after phase correction, (**b**) Phase noise (in unit of radians) before (black) and after (red) phase correction.

While SS-OCT offers the advantage in image speed, depth and SNR roll-off over camera-based SD-OCT, reducing scanning intervals may induce excessive phase noise for flow especially slow flow measurements for SS-ODT. Several factors, including mechanic stability, timing accuracy between laser sweeping and data acquisition, and the image SNR determine the system’s ability to resolve minute microvascular flows. The minimally detectable flow of ODT has been derived to be inversely proportional to system 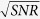
25,


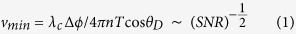


where Δ*ϕ* is the phase sensitivity, *λ*_c_ is the central wavelength, *T* is the time interval between sequential A-scans and *θ*_D_ is the angle between flow direction and the incident beam direction, and *SNR* is the shot-noise-limit SNR of a structural (OCT) image. It has been found^14^ that major phase errors of SS-ODT result from random phase shifts between adjacent A-scans due to the timing jitters between spectral sweeping of the swept laser and data acquisition. Moreover, as the phase shifts are linearly proportional to OPD, the phase errors are more severe in deeper image depths. Therefore, effective phase correction is critical for imaging flow, particularly microvascular flow in deep brain. In this design, a FBG filter is inserted between the laser and the interferometer so that it not only provides a stabilized A-scan clock (i.e., locked sweep trigger) but also maintains a balanced spectral intensity between the sample and the reference arms, which would otherwise disturb the interferogram detected by the balanced photo diodes near this wavelength. The Bragg wavelength of the FBG filter is specially designed to locate at the very edge (e.g., λ_B_ = 1270.4 nm) of the spectral-sweeping band. As the FBG is engaged in spectral-hopping mode, it blocks the incident light within λ_B_ = 1270~1270.4 nm (reflectivity = 99.91%) from entering the OCT engine, thus resulting in a near-zero output at the balanced detectors. Due to a semi-Gaussian sweeping profile of the laser, the measured interferometric signal at λ_B_ (close to the edge) is also close to zero, i.e., I(λ_B_) ≈0. Therefore, when this spectral impurity mixes with the subtle interferometric signal, it is still challenging to calibrate the interferograms with high accuracy solely based on either thresholding or tracking the falling slope of the FBG profile. To facilitate highly accurate phase correction, we implemented an interferogram registration method based on cross correlation, which can be described by the following algorithm,


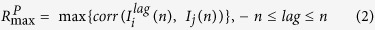



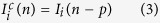


where *I*_*i*_(*n*) is the interferogram of the *i* th A-scan, lag is the relative shift between the *i* th and *j* th A-scans within the same B-scan. 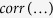 calculates the time-lagged correlation coefficient between the *i* th and *j* th A-scans and a 2*n* array *R* records the corresponding cross-correlation coefficients. After recursive calculation the maximum cross-correlation coefficient, 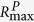, was derived with which the corresponding shift *p* was used to correct the interferogram 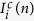 of the ith A-scan. Since this method circumvents the need for predefined threshold, it can be applied to any circumstance regardless of strong or weak surface reflection. Moreover, only very limited spectral range (*n* < 10) near λ_B_ needs to be searched in the process, the algorithm is computationally efficient and suitable for real-time display. To test the efficacy of this approach, a stationary mirror was used to characterize the phase noise at high SNR scenario (e.g., SNR = 106.3 dB) where the phase noise induced by light scanning and tissue inhomogeneity was excluded from the quantification. Figure 6a shows a snapshot of the overlapped interferograms from 1000 successive A-scans before and after phase correction. Without phase correction, the spectral phases among A-scans were severely washed out due to the fluctuation of timing-jitter (i.e., unstable sweep trigger). With phase correction, all of the interferograms were accurately realigned. Figure 6b shows that although the phase noise increased with OPD (i.e., image depth), the noise increase is substantially reduced by phase correction (red trace). If the phase noise Δ*ϕ* is defined as the standard deviation of phase differences across 4000 A-scans, Δ*ϕ* = 18 mrad at OPD = 500 um after phase correction is 73.5% lower than Δ*ϕ* = 68 mrad before phase correction. According to Eq.(1), the estimated theoretical flow detection limit is 268.2 μm/s with phase correction and 1013.2 μm/s without phase correction.

### Animal preparation

C57/B6 mice (male, 12–14 weeks of age, Jackson lab) were used to conduct the CBFv imaging. Mice were anesthetized with inhalational 2% isoflurane (in 100% O_2_) and mounted on a custom stereotaxic frame. The skull was thinned to transparent and covered by a 100 μm-thick glass coverslip with immunosuppressive drugs (dexamethasone sodium phosphate) applied. For cocaine study, a 100 μl bolus of cocaine (2.5 mg/kg, body weight) was rapidly injected (<15 s) through tail vein. All animal experiments were carried out according to National Institutes of Health guidelines and were approved by the Institutional Animal Care and Use Committee of Stony Brook University.

## Additional Information

**How to cite this article**: Chen, W. *et al*. High-speed swept source optical coherence Doppler tomography for deep brain microvascular imaging. *Sci. Rep.* **6**, 38786; doi: 10.1038/srep38786 (2016).

**Publisher's note:** Springer Nature remains neutral with regard to jurisdictional claims in published maps and institutional affiliations.

## Supplementary Material

Supplementary Information
