# Supplementary material for: High-speed swept source optical coherence Doppler tomography for deep brain microvascular imaging
Source: Sci Rep. 2016 Dec 9;6:38786. doi: 10.1038/srep38786 (PMC5146972; doi:10.1038/srep38786)
Supplement: Supplementary Information [file srep38786-s1.pdf]

Supplementary Information:

High-speed swept source optical coherence Doppler tomography for deep brain microvascular imaging

Wei Chen[1], Jiang You[1], Xiaochun Gu[1,2], Congwu Du[1], and Yingtian Pan[1,] *

[1]Department of Biomedical Engineering, Stony Brook University, Stony Brook, NY 11794, USA

[2]Key Laboratory of Developmental Genes and Human Diseases, Department of Anatomy and Neuroscience, School of Medicine, Southeast University, Nanjing 210009, China

[*]yingtian.pan@stonybrook.edu

Supplementary I:

Comparison in phase noise level between the pervious thresholding method and the proposed cross-correlation method

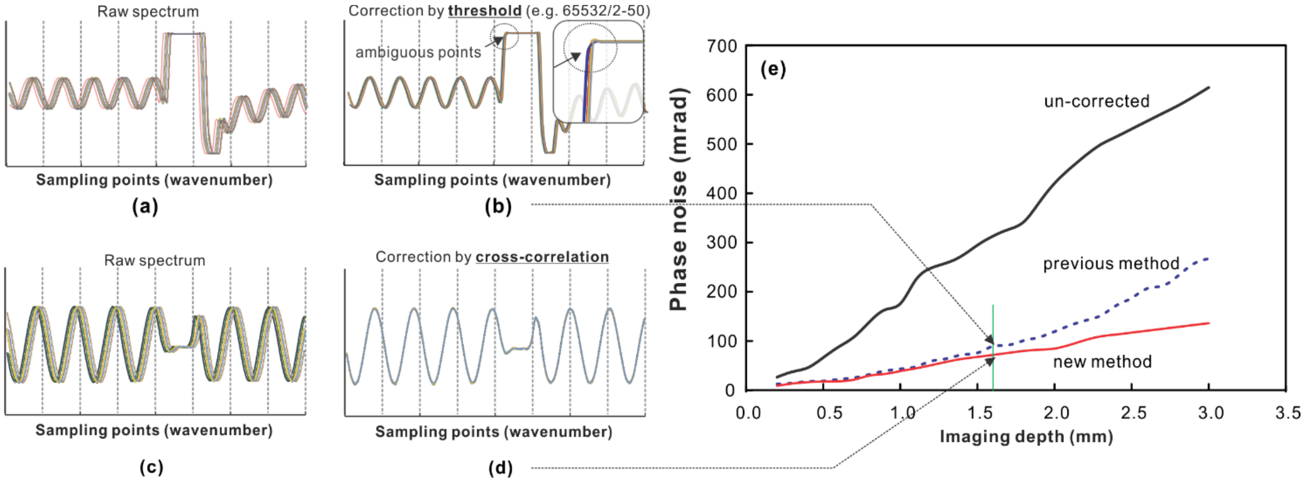

Fig. s1. (a-d) snapshots of the overlapped interferograms from 1k successive A-scans before and after phase correction using two different methods; (e) phase noise at different image depths. Red, dotted blue traces: residual phase noises after phase correction by previous and new methods; black trace: uncorrected phase noise.

The phase noise levels of the two methods were quantified by acquiring 4k A-lines per B-scan and 20 B-scans per image depth to assess the effects of image depth. For each depth, the phase noise $\Delta\phi$

was expressed as the standard deviation of the phase differences across 4k A-lines, and the mean phase noise ($\overline{\Delta\phi}$) is obtained by averaging across B-scans. Figs.s1(a-b) show the snapshots of the overlapped interferograms from 1k successive A-scans before and after phase correction using the previous method. Figs.s1(c-d) show the corresponding results using our cross-correlation method. The previous method (Fig.s1(b)) improves the phase stability of the original interferogram (Fig.s1(a)) by setting a threshold, e.g., at a saturation level at ~65535/2, but the phase errors after correction are noticeable. As the arrow in Fig.s1(b) points out, likely due to ambiguity for locking the rising edge, thresholding does not provide a clean cut and thus degrades the accuracy of phase correction. The new method shows drastically enhanced performance to realign the interference fringes (Fig.s1(d)), due to the tact that cross-correlation takes the entire FBG band (n=30-50 points) into consideration instead of one spot at the rising edge and is thus less vulnerable to random phase variation.

Fig.s1(e) plots the phase noise levels at difference image depths, which shows that the advantage of the new method is increasingly obvious with the increase of image depths. For instance, the phase errors of 18.2mrad (old) vs 16.7mrad (new) at 500μm of depth increase to 120.2mrad vs 84mrad at 2mm of depth. Overall, the new method is more robust for phase correction and the efficacy can be even more distinct in the presence of higher background noise (e.g., flow phantom, tissue vs mirror) as shown below in Fig.s2.

Supplementary II:

Comparison in flow sensitivity and noise background using flow phantom

A flow phantom study (a microvascular flow phantom using 0.5% intralipid in a 280μm ID micro tubing) was performed to compare the flow sensitivity and noise background between the two methods. Tests were performed at flow rates of v_p=0, 1.91, and 3.82 mm/s, which were controlled by a high-precision syringe pump. The SS-ODT images were obtained using two setups, i.e., FBG in reference arm for the thresholding method and FBG in the input arm for the cross-correlation method. In Figs.s2, upper (a-d) and lower (e-h) panels are the flow maps obtained by cross-correlation method and the previous method, respectively. Their flow velocity profiles in horizontal f(x) direction are plotted in panels (a'-d') and (e'-h'). At v_p=0 mm/s, the minimally detectable flows (i.e., flow noise floors) quantified by the cross-correlation method and by the previous thresholding method were 268μm/s and 421μm/s, respectively, as indicated by the dashed green lines in panels (a', e'). As the

flow rate increases, both methods started to show a parabolic flow distribution. However, due to background flow noise, the previous method presented lower flow contrast and more distorted parabolic flow profile (f'-h') than the new method (b'-d'). For a more quantitative comparison of SNR defined as SNR=v_{max}/v_{noise}, their differences were 8.6/3.8=2.26 (b'/f') for v_p=1.9mm/s, 19.2/6.9=2.78 (c'/g') for v_p=3.82mm/s, and 26.3/12.2=2.16 (d'/h') for v_p=5.76mm/s. These results of flow phantom study indicate the effectiveness of the new method for SNR improvement.

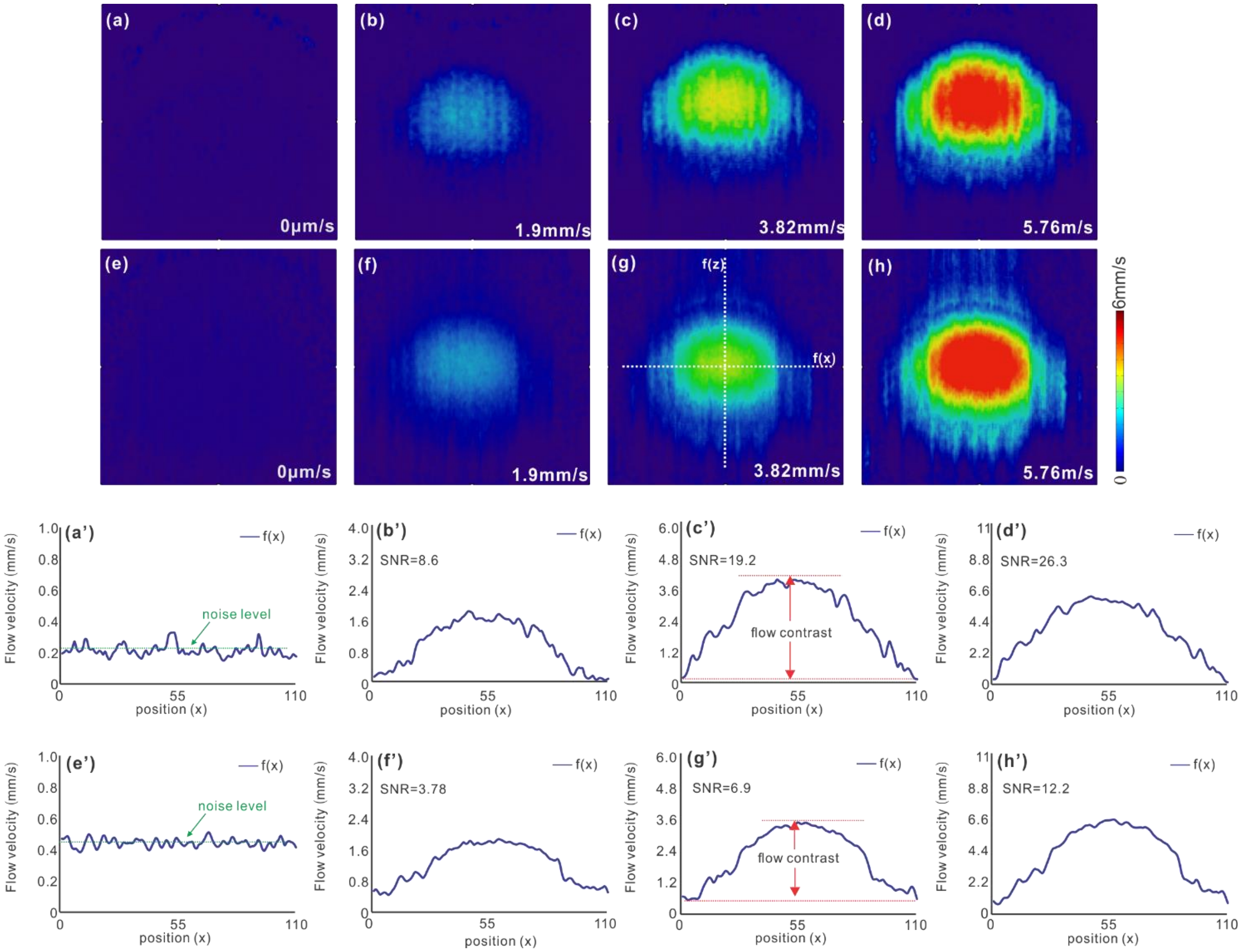

Fig. s2. (a-d) SS-ODT images acquired at different flow rates (v_p=0, 1.91, and 3.82 mm/s) with the FBG in the input arm for phase correction by cross-correlation; (e-h) the SS-ODT images acquired with the FBG in the ref arm for phase correction by thresholding. (a'-d') and (e'-h'): cross-sectional flow velocity profiles by the new method (a-d) and the previous method (e-h), respectively.
